# COVID-19 management at one of the largest hospitals in Germany: Concept, evaluation and adaptation

**DOI:** 10.1177/09514848221100752

**Published:** 2023-02

**Authors:** Ana Zhelyazkova, Philipp M Fischer, Nina Thies, Julia S Schrader-Reichling, Thorsten Kohlmann, Kristina Adorjan, René Huith, Karl-Walter Jauch, Stephan M Prückner

**Affiliations:** 1 Institute of Emergency Medicine and Management in Medicine, University Hospital, LMU Munich, Munich, Germany; 2 Department of Psychiatry and Psychotherapy, University Hospital, LMU Munich, Munich, Germany; 3 Department of Project Organization, University Hospital, LMU Munich, Munich, Germany; 4 LMU Munich, Munich, Germany

**Keywords:** COVID-19, healthcare management, pandemic preparedness, hospital organization, health care workers

## Abstract

**Context:**

The LMU University Hospital is among the largest healthcare facilities in Germany. The measures implemented prior to and during the first pandemic wave of COVID-19, were evaluated in preparation of a second pandemic wave. This paper presents the pandemic management concept, evaluation and adaptation of LMU University Hospital.

**Methods:**

Between July and September 2020 the disaster management team of LMU University Hospital conducted a mixed-method evaluation of the hospital’s pandemic management. A workshop series based on the After Action Review working group format was organized to examine the management structure, decision-making processes, documentation, and crisis preparedness response for a second COVID-19 wave. Further, the satisfaction of employees with the hospital’s COVID-19 management was examined through an anonymous survey.

**Results:**

The workshop series highlighted a need for structural and operational adaptation of the COVID-19 management at LMU University Hospital. The results of the employee survey (*N* = 2182) provided positive feedback for the measures taken during the first pandemic wave. Specific actions were derived concerning the availability of personal protective equipment and emergency childcare services. A key outcome of both evaluation activities was the identified need for further improvement in communication between stakeholders. All changes were adopted prior to the second pandemic wave.

## Background

On 11 March 2020, the World Health Organization (WHO) characterized the spread of SARS-CoV-2 as a pandemic^1^. On 16 March 2020, the Bavarian State Ministry of the Interior, for Sport and Integration declared a state emergency shifting the dynamics of healthcare services provision in the state.^2^

The LMU University Hospital is one of the largest healthcare providers in Germany. Affiliated with the medical faculty of the Ludwig-Maximilians-Universität, the hospital with its 28 specialized clinics rank among the top facilities for medical care as well as for medical research and teaching in the country (Table 1).^3^ The COVID-19 pandemic poses an increased challenge to the LMU University Hospital to provide high-quality healthcare to COVID- and non-COVID-patients while ensuring the infection control and safety of all employees.^4^ As early as 3^rd^ February 2020, the LMU University Hospital executive management began taking steps to strengthen the prevention of SARS-CoV-2 transmission at the hospital.^5^

**Table 1.** Overview of the capacity of LMU University Hospital and the number of treated COVID-19 patients.

LMU University hospital^1^	*n*
Personnel*	11,070
Physicians*	∼1800
Trained health care workers*	∼3300
Students (winter semester 2020/21)	7092
Patients (mean per year)	∼500,000
Full in-patient treatments*	69,764
1st wave of COVID-19 (03.-07.20)^2^	
All COVID-19 patients	222
ICU COVID-19 patients	81
Deceased COVID-19 patients	35
2nd wave of COVID-19 (09.20-02.21)^2^	
All COVID-19 patients	779
All ICU COVID-19 patients	163
Deceased COVID-19 patients	120
Total number of patients (01.02.2020 - 26.12.2021)^2^	
All COVID-19 patients	2071
All ICU COVID-19 patients	483
All deceased COVID-19 patients	266

*The information refers to the data from the year 2020.

^1^References for the data in table (the references are also made in the text)Klinikum der Universität München. Wir über uns. 2021; https://www.lmu-klinikum.de/das-klinikum/wir-uber-uns/e6d6f2726cf17b4e. Accessed 25.05.2021.

^2^CoPaMa Dashboard. Internal documentation; 2020. Access restricted to internal use only. Accessed 25.05.2021.

During the course of the pandemic, the hospital developed a comprehensive COVID-19 management structure. The measures taken were based on the internal guidelines on emergency preparedness and readiness, and were guided by the recommendations and regulations of the German Federal Office of Civil Protection and Disaster Relief, the European Centre of Disease Prevention and Control, and the Robert Koch-Institute.^6–9^ According to regular changes in policy regulations at the local, regional and national levels as well as due to the pandemic developments and the continuous increase of knowledge on SARS-CoV-2, the pandemic management at LMU University Hospital is subject to recurrent evaluation and adaptation.

This paper **aims** to provide an overview of the infection control and COVID-19 management concept of LMU University Hospital in Munich by presenting the measures taken during the first and second pandemic waves of COVID-19 as well as the perception of the implemented internal regulations by the hospital staff. After the evaluation, we present the pandemic management structure of LMU University Hospital as well as the implemented adaptations to it following the first and in preparation of the second wave.

## Methods

The evaluation process consisted of two main components implemented between July and September 2020. For the purposes of this paper, we define a “pandemic wave” as a noticeable increase in daily new cases and hospitalizations followed by an observable decrease of these two parameters. Consistent with trends in infection rates in Munich, Germany, we define the first pandemic COVID-19 wave as the period between March and July 2020, and the second pandemic wave as the period between September 2020 and February 2021.^10^ The definition corresponds to the retrospective classification of COVID-19 pandemic phases in Germany as defined by the Robert Koch-Institute.^11^

### After action review workshop series

The LMU University Hospital’s disaster management team conducted an After Action Review (AAR). The AAR was implemented following the working group AAR format developed by the WHO.^12^ The workshop participants corresponded to the different pillars of the pandemic management at LMU University Hospital during the first pandemic wave. The list of participants was based on the role of the represented departments as the main points of interaction between all stakeholders, which placed the essential focus of the workshops on intra- and inter-organizational interdependence.

The individual workshops were conducted as plenary discussions with the following main topics

**Figure 1:**
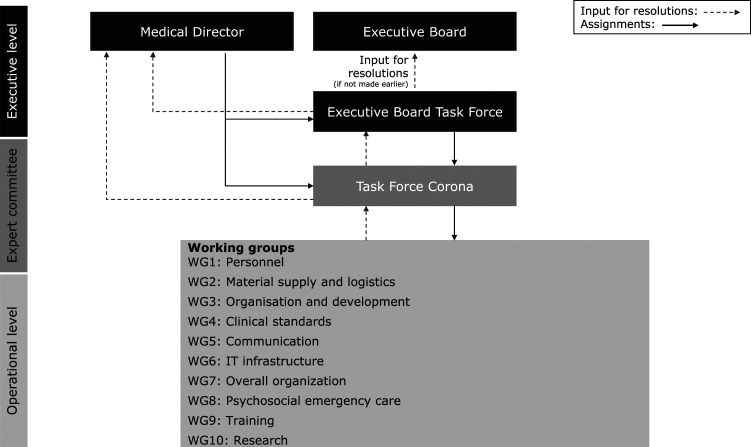
. [Originally adopted pandemic management structure of LMU University Hospital during the first pandemic wave of COVID-19 in Munich, Germany (approx. 03.-07.2020)].

**Figure 2:**
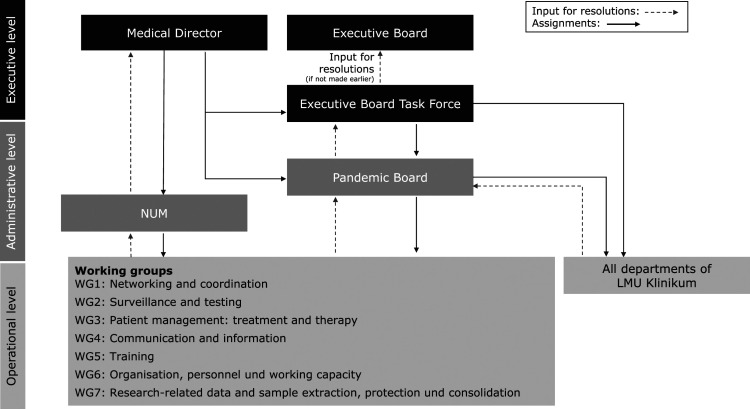
. [Pandemic management structure of LMU University Hospital during the second pandemic wave of COVID-19 in Munich, Germany (approx. 09.2020-02.2021)].

(1) Management structure and decision-making processes – identification of elements in need of optimization, challenges, and good practices arising from the originally adopted management structure (Figure 1);
(2) Document-Management-System (DMS) – review of the relevance and validity of all existing pandemic related documents (including standard operating procedures (SOPs), guidelines); development of a DMS for indexing and storage, collaboration and workflow, searching and retrieval; development of an internal Web site for publishing analogous to the defined structures
(3) Identification of specific measures to improve the preparedness prior to a second pandemic wave.

The workshop series, as well as the processing of the outcomes, were further informed by the relevant guidelines of the Federal Office of Civil Protection and Disaster Assistance and the European Centre for Disease Prevention and Control.^7,6^

### Employee survey

The LMU University Hospital disaster management team conducted an anonymous and voluntary survey to determine the satisfaction of employees with the adopted COVID-19 measures (Appendix 1). The questionnaire’s design was tailored specifically to the concerns and needs raised by the executive board and aimed at informing the further development of the pandemic management at the university hospital. During the designing process, no analogous employee surveys were identified, hence a reference could not be applied. The questionnaire underwent several rounds of revision by the team as well as pretesting by representatives of the Institute of the Emergency Medicine and Management in Medicine and the Department of communication, who were not directly involved in the topic.

The questionnaire was divided into five chapters: information and awareness (10 questions), LMU University Hospital measures (4 questions), professional environment (4 questions), personal environment (5 questions), and general and demographic data (8 questions).

The questionnaire was created using LimeSurvey (Version 4.3.3 + 200,707). All employees received an e-mail invitation to participate in the survey and received regular reminders through the intranet page and the COVID-19 newsletter. For employees without access to a workplace computer, a printed version was created using the program Zensus direkt (Blubbsoft GmbH). The ethics committee of the medical faculty of the University of Munich approved the study (Project-No 20-614 KB).

The online version was available to all employees from 16.07.2020 until 06.08.2020. The printed questionnaires were delivered to the respective departments on 23.07.2020. The completed and sealed questionnaires were collected on 04.09.2020. The analysis was carried out with IBM SPSS Statistics Version 26 software package. We tested the interaction between participants’ characteristic and their satisfaction with the adopted measures using Spearman-Rho and Phi coefficients. A subgroup analyses was conducted using the Mann-Whitney-U-Test as well as the Jonckheere Trend-Test depending on the data format.

## Results

### After action review

Three face-to-face workshops were conducted between 10.07.2020 and 09.09.2020. The number of participants varied between nine and 12 people at each workshop. All participants received a detailed results report. Since the documentation was designated for internal use only, we provide a brief summary of the key outcomes in the following section. Two main aspects requiring optimization emerged from the discussions.

The first workshop (10.07.2020) was focused on compiling an overview of the participants’ impressions of the management structure and decision-making processes during the first wave. The participation was purposefully limited to the disaster management team and one representative each of the executive board, of the department of project management and of the department of organizational development as to strategically inform the topical organization of the further workshops (*N* = 7). The main problematic area identified within the workshop was the need for a more precise definition of the tasks, the teams, and the individuals responsible for those. Participants identified this as a cause of inadequate communication between departments and the stakeholders within and between the executive and operational levels of the COVID-19 response. Participants acknowledged that the suboptimal delegation of responsibility originated from uncertainties regarding the virological, medical, and epidemiological properties of SARS-CoV-2. The problem was reflected in the lack of description of the scope of duties expected to be covered by the working groups in the first wave.

The second workshop (03.08.2020) targeted a more implementation-oriented aspect of improving the communication between stakeholders by streamlining the documentation management. Participants included representatives the department of communication, human resources, project management and organizational development (*N* = 7). The participants underscored the need for a project management platform that categorizes, regularly updates, and communicates all documents related to the hospital’s pandemic response management. A special need was identified in updating or removing outdated regulations that have changed due to improved knowledge or to changes in legal measures by the public health policy-makers and institutions. Another advantage of having a platform over the method of individually uploading individual documents to the designated intranet site used at the time was seen in the consistent availability and convenience of communication to staff. In addition, participants suggested that for the platform to act as the point of access for all administrative documentation of executive meetings, including agendas, protocols, and presentations.

The third and final workshop focused on compiling and discussing the thus far collected input into a strategically laid-out management structure for the new Pandemic Board. Participants included all previously involved stakeholders (*N* = 11). The workshop served as a final feedback round prior to the first meeting of the Pandemic Board on 10.09.2020. All outcomes are presented in Adaptation of pandemic management structure section.

### Employee survey results

Out of 11 070 employees of the LMU University Hospital, 3139 participated in the online survey, and 2182 questionnaires were completed. Only completed questionnaires were analyzed (Table 2). The majority of questions were designed with a 5-point Likert scale with options ranging between “strongly disagree” at item one and “strongly agree” at item 5.

**Table 2.** Demographic and occupational data of surveyed employees.

	*N*	%	Coefficient*	*p*-value
Age				
<20 years	9	0.40	0.100**	.001
20-30 years	385	17.60	—	—
30-40 years	519	23.80	—	—
40-50 years	462	21.20	—	—
50-60 years	586	26.90	—	—
>60 years	169	7.70	—	—
No answer	52	2.30	—	—
Occupation				
Nonmedical staff	1062	48.70	0.100***	.000
Medical staff	1120	51.30	—	—
Regular home office				
Yes	415	19.00	0.119***	.000
No	864	39.60	—	—
Home office not possible	807	37.00	—	—
No answer	96	4.40	—	—
Work with COVID-19 patients				
Yes	197	9.00	0.106***	.001
Mean number of weeks = 8.20 (SD = 5.60)				
No	1985	91.00	—	—
Rotation to a different unit				
Yes	131	6.00	0.064***	.073
No	1967	90.10	—	—
No answer	84	3.80	—	—
All	2182	—	—	—

The survey results regarding the occupational location, primary units of service and rotational units of service is not reported here as the information is designated for internal use only. *Coefficients were measured for the employees’ satisfaction with the measures adopted by the LMU University Hospital. **The correlation was measured with Spearman-Rho as both variables are ordinally scaled. ***The correlation was measured with the Phi Coefficient as the independent variables are nominally scaled.

Overall, the participants felt well informed by the hospital management during the first wave (8 items; α = 0.875). Access to the relevant information on the intranet (mean = 4.19; SD = 1.02) and in the IT portal (mean = 4.03; SD = 1.04) was perceived positively. The information provided by the LMU University Hospital on the current situation (mean = 4.03; SD = 1.03) and the measures taken (mean = 4.01; SD = 1.04) on various platforms was generally rated as good: newsletter “News from the Executive Board Task Force” (mean = 4.38; SD = 0.84), information on the intranet (mean = 4.00; SD = 1.02), and on the IT portal (mean = 3.92; SD = 1.02), information from superiors (mean = 3.52; SD = 1.34). In terms of additionally desired communication channels, posters (mean = 2.06; SD = 1.15), flyers (mean = 2.00; SD = 1.13) and videos (mean = 2.08; SD = 1.12) were not required (4 items; α = 0.699). The development of an employee app was perceived as a good idea by the majority of participants (mean = 3.60; SD = 1.45).

#### Measures of the LMU University Hospital

The subscale consisted of 4 items (α = 0.771). The measures of the LMU University Hospital were considered sufficient by the respondents (mean = 4.00; SD = 1.10). The measures in the respective departments (i.e. individual clinics, units) were also rated as sufficient (mean = 3.68; SD = 1.31). The scarcity of personal protective equipment (PPE) at the beginning of the first wave is well noted in the result of the corresponding question (mean = 2.96; SD = 1.45). However, the improved availability of PPE as the pandemic progressed is also reflected in the survey results (mean = 3.76; SD = 1.24).

#### Work and personal circumstances

Overall 4 work-related items were tested (α = 0.528). The participants tended to disagree with the statement that their work duties had changed significantly since March (mean = 2.37; SD = 1.35). Participants' workloads have not significantly decreased (mean = 1.73; SD = 1.01); conversely, however, they also did not clearly report an increased workload (mean = 3.13; SD = 1.43). Perceptions of team spirit in own´s department were not elevated (mean = 2.77; SD = 1.24).

Two items tested the concern of infection (α = 0.630). The majority of participants had no fear of contacting COVID-19 infected individuals at the work place (mean = 2.66; SD = 1.40) with slightly lower concern about infection in a private setting (e.g. family) (mean = 2.30; SD = 1.18).

In terms of childcare, 24.5% (*n* = 507) of participants reported having children of young age. 14.8% (*n* = 307) were parents of children attending day care or kindergarten and 9.7% had children attending school. Parents were further asked to evaluate the childcare management of the hospital (2 items; α = 0.846). Most parents felt inadequately informed about emergency childcare services at LMU University Hospital (mean = 2.62; SD = 1.35) after schools, kindergartens and day care centers closed. They also felt that the emergency childcare services taken by the LMU University Hospital were not satisfactory (mean = 2.54; SD = 1.31).

#### Subgroup analyses

Based on the effects presented by the variables in Table 2, we executed subgroup analyses for the satisfaction with the measures adopted by the hospital (Table 3).

**Table 3.** Subgroup analyses for the satisfaction of employees with the general information provision of the LMU University Hospital.

Satisfaction with the measures adopted by the LMU university hospital	Median	Mean ± St.Dev	τb	*p*
Regular home office*			−0.087	.000
Yes (*n* = 415)	4.00	4.15 ± 0.958	—	—
No (*n* = 864)	4.00	4.02 ± 1070	—	—
Home office not possible (*n* = 807)	4.00	3.83 ± 1176	—	—
Age**			0.081	.000
<20 years (*n* = 9)	5.00	4.33 ± 0.866	—	—
20–30 years (*n* = 385)	4.00	3.85 ± 1146	—	—
30–40 years (*n* = 519)	4.00	3.98 ± 1082	—	—
40–50 years (*n* = 462)	4.00	3.96 ± 1141	—	—
50–60 years (*n* = 586)	4.00	4.14 ± 1023	—	—
>60 years (*n* = 169)	4.00	4.08 ± 1069	—	—
Satisfaction with the measures adopted by the LMU university hospital			*r*	p
Work with COVID-19 patients***			0.069	.001
Yes (*n* = 197)	4.00	3.70 ± 1276	—	—
No (*n* = 1985)	4.00	4.02 ± 1072	—	—
Occupation***			0.098	.000
Nonmedical staff (*n* = 1062)	4.00	4.11 ± 1043	—	—
Medical staff (*n* = 1120)	4.00	3.89 ± 1134	—	—

*Kruskal-Wallis *p*-value = 0.000; the post-hoc test was executed using Jonckheere Trend-Test at *p* = 0.000; the correlation effect was calculated with Kendall-Tau-b. **The option “No answer” was excluded from this analysis, as it does not provide any insights into the correlation. Kruskal-Wallis *p*-value = 0.001. ***The analysis was executed with a Mann-Whitney-U-Test as the variable is binary. On the scale of 1 to 5 where “strongly disagree” is placed at item one and “strongly agree” at item 5.

There were significant yet very weak differences based on the ability to work from home during the pandemic with those who do not or are not able to do so evaluating the adopted measures as less sufficient than those who do work from home (τb = −0.087; *p* = 0.000). The opposite effect was observed with age, where the older the participants were the better they perceived the measures adopted by the hospital (τb = 0.081; *p* = 0.000). The effects of working with COVID-19 patients and of the occupational area of participants were similarly very weak (r = 0.069, *p* = 0.001; respectively r = 0.098, *p* = 0.000).

### Adaptation of pandemic management structure

All first wave measures were de-escalated, re-escalated, and qualitatively adjusted depending on the current pandemic and the hospital activity in Munich and Bavaria. Changes to the applied measures were communicated via the newsletter as well as the designated intranet page “Update Coronavirus”. A detailed report with the results of the employee survey and management recommendations derived from it were made available to all employees.

A number of specific aspects of pandemic management at the LMU University Hospital requiring optimization were derived from the results of the workshop series as well as of the employee survey. The adjustments adopted prior to the second pandemic wave in Munich, between September 2020 and February 2021, are described below.

#### Management structure

In preparation for an expected second wave in the fall of 2020, adjustments in the management structures were adopted (Figure 2). A new management level, represented by the Pandemic Board, was implemented in order to address the main topics of concern highlighted through the workshops. The Pandemic Board was primarily tasked with improving the communication between stakeholders as well as towards employees by

• Coordinating the information flow between the executive and operational levels;
• Handling pandemic related inquiries from internal and external stakeholders including questions from employees;
• Conducting regular meetings with executive and operational level representatives during periods of lower pandemic activity (during less acute phases of incidence rates when Executive Board Task Force meetings are not necessarily required on a regular basis).

Additionally, the thematic focus of the working groups was laid out in detail to better reflect the interdisciplinary nature of the teams needed to effectively manage the intra-hospital response. In order to ensure the effective delegation and tracing of tasks, the Pandemic Board compiled a profile for each working group reflecting the spectrum of responsibilities that the group is expected to manage. (Appendix 2)The adjustment also resulted in a reduction in the number of working groups, facilitating communication among all stakeholders. Furthermore, the Pandemic Board compiled a Pandemic Response Plan incorporating an exhaustive task assignment covering the complete organizational structure of the hospital (including individual departments outside of the Boards’s jurisdiction).

The Task Force Corona operating during the first wave was dissolved in its role as an expert committee. The experts involved were accordingly included in the adapted structure of the working groups and were regularly invited as expert advisors to the meetings of the Executive Board Task Force.

The adaptations of the management structure were further informed by the Nationales Netzwerk Universitätsmedizin (NUM; National Network University Medicine). NUM was established in March 2020 with the aim of facilitating a coordinated COVID-19 response among all German university hospitals.^13^

#### Document-management-system

For the efficient handling, updating and publishing of all pandemic related documents, a DMS according to the DIN EN ISO 9001:2015 quality standard was implemented. Starting June 2020 and prior to the launch of the platform, the Project Organization Department conducted a detailed content evaluation of all COVID-19-related documentation generated within the LMU Klinikum during the first wave. A total of 294 documents were identified. Out of these, 138 documents had to be adapted to the currently valid regulations (content, structure, indexing). In September 2020, the DMS was launched analogous to the defined structures of the new management structure.

#### Communication

The DMS platform further facilitated the communication of the latest documentation to all hospital employees. By connecting the DMS platform to the intranet site on COVID-19, the system provided faster access to the latest versions of all documents shortly after the conclusion of the latest updates.

Following the results from the employee survey, the dedicated COVID-19 intranet site and the regular newsletter were established as the main media to inform employees about the latest updates. The newsletter underwent a minor rebranding process to better reflect the diversity of sources and information, and was therefore renamed “News on the Corona pandemic”. In addition, the implementation of a mobile application for employees was identified as a crucial step to further improve internal communication. The development of a mobile application for internal use is now in the planning stage.

#### PPE availability

In addition to ensuring increased availability of PPE in the workplace, PPE products were also offered for purchase for private use. All products offered were tested by the LMU University Hospital´s Department of Clinical Microbiology and Hospital Hygiene for compliance with the certification requirements. The offer was particularly well received by employees before the Christmas holidays and when FFP2-mask requirement came into force in Bavaria starting 18 January 2021.^14^

#### Childcare services

The human resources department and the Executive Board Task Force contracted an additional provider to extend the emergency childcare services to all employees with children attending educational institution up to the age of 6, and for the children with special needs. Communication of the services was intensified to ensure a better awareness of the measures.

#### Employee psychosocial support

Given the ongoing status of the COVID-19 pandemic and the resulting need for long-term implementation of pandemic management, the LMU University Hospital aims to expand and intensify measures to provide psychosocial support for all employees. Within the frames of the longitudinal Care Corona Immune Study (CCI; 4 study visits; *N* = 220) and the cross-sectional All Corona Care Study (ACC; *N* = 7546) performed towards the end of the first wave, we conducted several epidemiological psychosocial analyses.^15,16^

We applied a machine-learning framework to facilitate single-subject predictions of pandemic-related psychological distress in healthcare workers. We used a cross-over design to investigate the predictive value of epidemiological and psychological variables in the populations of two studies. Our ultimate aim was to develop a simple tool for predicting pandemic-related psychological distress in individual healthcare workers and to build specific, generalizable models with the potential to inform clinical decisions. To achieve this, we also aimed to examine new variables with predictive value and to stratify employees therapeutically to allow us to offer interventions to relieve psychological distress. To our knowledge, this is one of the first precision tools in psychiatry to adopt a dual, i.e. clinical and research approach. If the predictive tool is found to be successful, in a future step we aim to implement it in clinical settings. A publication with detailed results of the analyses is forthcoming.

In line with these preliminary results as well as with the designation of 2021 as *International Year of Health and Care Workers,* further measures targeting resilience-building and psychosocial support for employees are currently in development.^17^

## Discussion

In order to improve the pandemic management of the LMU University hospital, we conducted a mixed-method evaluation of the measures and structures adopted during the first wave of COVID-19. We conducted an AAR with representatives of the management level to assess the decision-making process and derive specific measures for their structured adaptation to fit better the needs of the hospital and its employees. Further, we conducted an anonymous and voluntary employee survey to ascertain the hospital employees’ satisfaction with the adopted measures during the first wave. The results demonstrated the general appropriateness of the response management, but also showcased how establishing and coordinating an effective management structure to efficiently support executive-level decision-making and communication to all employees is the crucial component that needed to be revised.

A DMS as lean, simple, and efficient as possible to support the timely management and internal communication of pandemic related documentation among stakeholders at the management, administrative and operational levels. Additionally, a critical aspect that emerged from the evaluation process was communication and support for employees. The subsequently extended availability of emergency childcare services and PPE (including for personal use) has been well received. Further, the results from the subgroup analyses demonstrated a relatively consistent satisfaction with the adopted measures, this indicating that a holistic communication approach inclusive of all employee groups would be beneficial.

The response to the COVID-19 pandemic highlighted the difficulties of establishing a comprehensive management structure against the background of lacking decisive estimates of the time, personnel, and infrastructure resources needed. Uncertainties regarding the virologic, medical, and epidemiologic characteristics of SARS-CoV-2 required the rapid establishment of transdisciplinary teams that would however be expected to operate efficiently over an unpredictably long period. Thus, while immediate response and patient management were highly profitable in the first pandemic wave, management decisions made in March of 2020 required adaptation based on the systematic implementation of organizational governance.

### Limitations

Beyond February 2021, LMU University Hospital’s overall pandemic management and individual measures continue to be a subject of regular evaluation. The evaluation process of the LMU University Hospital response and management after the first COVID-19 wave in Munich remains limited due to several factors. While the AAR implemented working group format allowed for open and honest discussion of optimization needs, planning and executing this format presented a great organizational challenge especially under the circumstances limiting in-person meetings. Consequently, the disaster management team was required to organize the review during a response that was still technically underway even though it was in a relatively moderate phase compared to period of incidence peak in April of 2020.^10^ The suboptimal timing could have potentially led to misperceptions of the relevance, key findings, or functionality of various elements of the management structure. It is essential to note that the questionnaire contents were mainly driven by the necessity for internal evaluation commissioned by the executive board, hence there are no direct references to the presented outcomes. Furthermore, the employee survey reached approx. 1/3 of LMU University Hospital employees, which limits the internal and external generalizability of the results. Nevertheless, the outcomes of the evaluation process provide a general overview of the main topics, concerns, and challenges of establishing and coordinating a COVID-19 response in a large hospital.

The decision-making processes were and still are dependent on the latest knowledge of SARS-CoV-2 as well as on the incidence rates and their development within and beyond administrative and geographical borders. Nevertheless, it is evident that the internal hospital pandemic management and continuous optimization efforts adopted by LMU University Hospital are consistent with nationally recommended best practice models and leadership strategies for COVID-19 management as well as with the existing evidence on constructing a people-centered approach in the healthcare workforce governance.^18,19^

Beyond February 2021, LMU University Hospital aims to further improve its internal COVID-19 management by increasing efforts in the regular evaluation of adopted measures. Highlighting employee appreciation and enhancing employee wellbeing through improving the workplace atmosphere outline the frame of the hospital’s priorities for 2021.

### Implications for research and practice

The presented analysis differs in purpose and execution from previously published and standardized methods of analysis of employee satisfaction as it addresses a bottoms-up need for assessment of an acute situation rather than of the general working context and satisfaction. Nonetheless, it provides insight into how to approach several of the aspects previously identified as particularly influential on employee satisfaction: managerial structures and participation in decision-making processes.^20^ Despite the narrow scope of the evaluation, the results correspond to previously identified challenges to hospital management especially in the realm of value streaming processes.^21^

Our analysis highlights the requirements for implementing a structured, agile and people-centered pandemic management in a hospital setting. The implementation process calls for responsive decision-making generating an exhaustive spectrum of measures that take into account the personal challenges of stakeholders, especially employees, beyond the realm of the workplace. The results provide empirical evidence for the evaluation of internal organizational dimensions in maturity models in a hospital context (structure, management, decision-making, people management).^22^ Furthermore, the specific constraints of the topical scope and timeline of the this paper add insights into the evaluation of medical care provision in times of crisis beyond the implementation of field hospitals and within the framework of an already existent and functioning healthcare organization and system.^23,24^

The broad range of involved entities in this context requires the adoption of a continuous, self-critical, and flexible evaluation process. As the COVID-19 pandemic meets the definition of a *wicked problem*, investigative and systematic approaches towards its solving have been increasingly diverging from the design of classical emergency management systems.^25,26^ Instead, pandemic management approaches and interventions observed since March 2020 in diverse contexts worldwide are rather informed by the concepts of co-production and inclusion.^27–29^ The short and long-term consequences of co-production initiatives in healthcare systems and stakeholders remain to be explored beyond COVID-19.

The successful implementation of preventative measures in a hospital setting assumes open and honest communication between all stakeholders. This inevitably highlights any intra- and inter-organizational interdependences within the healthcare facility as well as within the area of regional healthcare services. The process calls for the full disclosure of uncertainties and of the characteristics and issues of the decision-making processes leading to the adoption of measures. While there is a large body of theoretical and empirical evidence on inter-organizational collaboration in a hospital context, thus supporting the planning and execution of efforts, more research is needed on hospital intra-organizational partnerships and networks.^30^ Our analysis serves as an exploratory case study of intra-organizational collaboration, yet further systematically obtained evidence is required.

## Conclusion

The presented analysis provided insights into the relevance of intermediate evaluation of managerial structures and decision-making processes during COVID-19. We examined the functionality of the executive structure in place during the first wave of COVID-19 in one of the largest hospitals in Germany as well as the satisfaction of the hospital’s employees with the catalogue of adopted measures. Our findings suggest that the executive structures and communication tools of intra-organizational collaboration are critical to the functionality and effectiveness of the pandemic response.

## Appendix 1 Overview of the infection protection and pandemic management measures at LMU University Hospital.

**Table:**

Category	Measure	Period of validity*		Adjustment during periods of lower pandemic activity
		1st wave (03.-07.20)*	2nd wave (09.20-02.21)	
Work organization and management	Maximum reduction of physical meetings; limited categories of in-person meetings and events allowed upon confirmation of mandatory adherence to the provided hygiene plan of the LMU university hospital (e.g. essential trainings, meetings of the executive board task force)	Yes	Yes	No
	Temporary suspension of elective and non-emergency patient admission (for patients and procedures allowing postponement of 6 months or longer without any health risk or disadvantages for the patient)	Yes	Yes	Yes
	Implementation of telemedical consultation services in lieu of in-person consultations	Yes	Yes	Yes
	Maximum reduction of business trips, prohibition of business trips in risk areas	Yes	Yes	No
	Prohibition of team gatherings and excursions	Yes	Yes	No
	Additional recruitment of students of human medicine and nursing for providing support in inpatient care	Yes	Yes	Yes
	Adapted regulation for inner hospital resuscitation to include the necessary infection protection including the deployment of “COVID-19 sets” for inner hospital resuscitation	No	Yes	No
Employee management	Temporary home office working arrangement for all employees and workspaces allowing the fulfillment of essential responsibilities through home-based teleworking	Yes	Yes	Yes
	Extended provision of emergency on-site childcare services for employees	Yes	Yes	Yes
	Adaptation of duty schedules due to the reduction of workload in individual wards (rotation of personnel to wards with increased workload, especially COVID-19 wards)	Yes	Yes	Yes
	Psychosocial support services for employees, patients and patients’ family members	Yes	Yes	Yes
	- Patient hotline #WeAreHereForYou (”#WirsindfürSieda”)			
	- Psychosocial support for COVID-19 patients via phone or digital platform in COVID-19 hospital wards			
	- Psychosocial support for COVID-19 patients’ family members			
	- Psychosocial support hotline for employees #HelptheHelperLMUKlinikum			
Equipment	Offering employees to purchase certified medical masks for private use (including FFP2 masks) via the hospital’s suppliers	No	Yes	No
	In-house testing and certification of PPE (especially FFP2 masks)	Yes	Yes	No
Training	Training material (video, posters) on the correct handling of personal protective equipment (PPE) available in 11 languages	Yes	Yes	No
	On-site trainings on the correct handling of PPE	Yes	Yes	No
	Additional trainings in ICU patient and emergency management, emergency caesarean section in COVID-19 patients	Yes	Yes	Yes
Information & communication	Several online events for employees providing the latest information on COVID-19 as well as on the measures adopted by LMU university hospital including Q&A sessions (events held in Mar, Oct, Dec 2020)	Yes	Yes	No
	Video podcast series with executive board members and senior staff members of the LMU university hospital addressing the current information needs of employees (e.g. testing procedures)	Yes	Yes	Yes
	Newsletter providing information on the latest adopted resolutions as well as updates on the pandemic situation in the hospital, in Munich and the surrounding area (frequency depending on the pandemic situation – from daily to bi-weekly)**	Yes	Yes	Yes
	Intranet page „update coronavirus“ as a single-stop platform for all information and internal documents on the topic of COVID-19	Yes	Yes	No
	Development of an IT-portal as a single-stop platform for all information and internal documents on the topic of COVID-19 accessible outside of the hospital network	Yes	Yes	No
	Continuous information campaign on the current hygiene and visiting regulations on all sites of the LMU university hospital (poster, videos, flyer, internet page) for patients, visitors and staff members	Yes	Yes	No
Infrastructure	Admission control checkpoints with integrated disinfection dispensers and provision of medical masks	Yes	Yes	Yes
	Separate entrance and exit points for employees and for patients/visitors	Yes	Yes	No
	Mandatory registration of all patients and visitors entering any of the hospital sites including a short questionnaire on symptoms of and exposure to COVID-19	Yes	Yes	No
	Increasing the availability of parking spaces for employees thus facilitating the use of private vehicles instead of public transportation	Yes	Yes	Yes
	Temporary adjustment of food supply in the hospital’s canteens to offer to-go meals only	Yes	Yes	Yes
	Limited sitting arrangements in all rooms incl. Cafeterias and waiting areas at all sites of the LMU university hospital	Yes	Yes	Yes
	Increased availability of disinfection dispensers in all sites of the LMU university hospitals	Yes	Yes	No
Infection protection & security	A general ban on visiting patients with the following exceptions: Visitors of patients in palliative care or dying patients; person accompanying a pregnant woman during birth (e.g. midwife); father visiting the newborn and its mother; parent or legal guardian of a sick child under 18 years of age; for urgent medical reasons (in consultation with the ward’s senior physician)	Yes	Yes	Yes
	Mandatory face masks at all sites of the hospital for patients, visitors and staff members including in departments without patient contact (e.g. administration, research facilities)	Yes	Yes	No
	Increased security personnel for monitoring and facilitation of the adherence to the pandemic measures	Yes	Yes	No
Testing	Algorithm for preoperative PCR-testing of all patients	Yes	Yes	No
	Standard operating procedure for PCR-testing of employees	Yes	Yes	No
	- Employees working in high-risk wards (e.g. ICU, emergency, labor wards)			
	- Employees returning to work after traveling (double-test principle)			
	- Within the frame of inner hospital outbreak management			
	- All employees wishing to get tested			

*all measures have undergone a continuous process of revision and adaptation in accordance with the current pandemic situation in Munich and the surrounding area as well as with the latest scientific evidence. **Between the first and second pandemic wave the newsletter has been rebranded and renamed from “News from the crisis committee of the executive board” (“Aktuelles aus dem Krisenstab des Vorstands”) to “News on the Corona pandemic” („Aktuelles zur Corona-Pandemie”).

## Appendix 2 Overview of the spectrum of responsibility of each working group after adjusting the management system.

**Table:**

Working group	Spectrum of responsibilities	Management*
WG1: Networking and coordination	Coordination of the exchange with external partners on local and regional level (e.g. the health department of the city of Munich, the disaster management officers on local, regional level)	Disaster management officer of the LMU university hospital Chair of the NUM COVID-19 task force at the LMU university hospital
	Coordination with the nationales netzwerk Universitätsmedizin (NUM)	
	Operational oversight of the working groups	
	Strategical oversight and support of the hospitals pandemic management	
	Strategical oversight and support of the COVID-19 related research activities of the hospital	
WG2: Surveillance and testing	Coordination of the testing possibilities and centers within the hospital	Chair of department of virology and Max-von-pettenkofer institute at Ludwig-Maximilians-Universität Chair of the department of clinical microbiology and hospital hygiene at the LMU university hospital
	Coordination of the measures for preventing outbreaks within the hospital	
	Compilation of SOP’s for managing positively tested personnel as well as outbreaks within the hospital	
WG3: Patient management: Treatment and therapy	Coordination of the capacities for treating infectious and non-infections COVID-19 patients incl. Facilitating escalation and de-escational processes regarding capacity managemet	Director of the clinic for anesthesiology Senior physician of the internal intensive care unit with focus on gastroenterology and pneumology
	Compilation of SOP’s for treating COVID-19 patients in different stages of the disease	
	Coordination of the palliative care of COVID-19 patients (incl. Triage if needed)	
WG4: Communication and information	Coordination of the communication of up-to-date information to all employees of the hospital	Director of the department of communication of the LMU university hospital Senior physician in clinical infectiology
	Operational support of the implementation of the new DMS	
	Managing external COVID-19 related inquires by media	
WG5: Training	Compiling training standards for the use of PPE	Senior physician in anesthesiology, MME-trained (Master of Medical Education) Head of the staff development and nursing science unit
	Coordinating and providing the training programs for PPE within the hospital (incl. Posters, videos)	
	Coordinating and providing training for the newly employed personnel especially students of human medicine	
WG6: Organisation, personnel und working capacity	Coordination of the infrastructure of the hospital (incl. Access control and regulations, organization of the sanitation measures)	Director of the department of strategic corporate management
	Coordination of the measures regarding personnel (incl. Personnel rotation, home office regulations, childcare support)	Co-Chair of the clinic for psychiatry and psychotherapy
		Department of project management
WG7: Research-related data and sample extraction, protection und consolidation	Compilation and report of the newest research on COVID-19	Chair of medical clinic 3 (focus on hematology and oncology) at the LMU university hospital
	Compilation and management of the data of all patients treated in the hospital	Chair of the department for biomedical informatics at the Ludwig-Maximilians-Universität

*All managers of the working groups we able to coordinate the constellation of the working groups themselves.
